# The Principles of Buoyancy in Marine Fish Eggs and Their Vertical Distributions across the World Oceans

**DOI:** 10.1371/journal.pone.0138821

**Published:** 2015-10-14

**Authors:** Svein Sundby, Trond Kristiansen

**Affiliations:** Institute of Marine Research and Hjort Centre for Marine Ecosystem Dynamics, P.O. Box 1870 Nordnes, 5817, Bergen, Norway; National Oceanic and Atmospheric Administration/National Marine Fisheries Service/Southwest Fisheries Science Center, UNITED STATES

## Abstract

Buoyancy acting on plankton, i.e. the difference in specific gravity between plankton and the ambient water, is a function of salinity and temperature. From specific gravity measurements of marine fish eggs salinity appears to be the only determinant of the buoyancy indicating that the thermal expansions of the fish egg and the ambient seawater are equal. We analyze the mechanisms behind thermal expansion in fish eggs in order to determine to what extent it can be justified to neglect the effects of temperature on buoyancy. Our results confirm the earlier assumptions that salinity is the basic determinant on buoyancy in marine fish eggs that, in turn, influence the vertical distributions and, consequently, the dispersal of fish eggs from the spawning areas. Fish populations have adapted accordingly by producing egg specific gravities that tune the egg buoyancy to create specific vertical distributions for each local population. A wide variety of buoyancy adaptations are found among fish populations. The ambient physical conditions at the spawning sites form a basic constraint for adaptation. In coastal regions where salinity increases with depth, and where the major fraction of the fish stocks spawns, pelagic and mesopelagic egg distributions dominate. However, in the larger part of worlds’ oceans salinity decreases with depth resulting in different egg distributions. Here, the principles of vertical distributions of fish eggs in the world oceans are presented in an overarching framework presenting the basic differences between regions, mainly coastal, where salinity increases with depth and the major part of the world oceans where salinity decreases with depth. We show that under these latter conditions, steady-state vertical distribution of mesopelagic fish eggs cannot exist as it does in most coastal regions. In fact, a critical spawning depth must exist where spawning below this depth threshold results in eggs sinking out of the water column and become lost for recruitment to the population. An example of adaptation to such conditions is Cape hake spawning above the critical layer in the Northern Benguela upwelling ecosystem. The eggs rise slowly in the onshore subsurface current below the Ekman layer, hence being advected inshore where the hatched larvae concentrate with optimal feeding conditions.

## Introduction

The transport and dispersion patterns of fish eggs and larvae during the early life stages of fish were hypothesized by Hjort [1] to be critical for the formation of year-class strength. These physical processes can be divided into two different components:

1. the mean ocean circulation pattern including the average tidal mixing that is part of the “ocean circulation climate”; these are the stabilizing factors to which the fish population can adaptively respond to maximize their reproductive success, and
2. the variable and stochastic “weather” of the ocean circulation that deviates from the average pattern induced by the variability of atmospheric forcing, transient eddy formation, and the initiation of surface and internal wave pattern. Together with the variability in abundances of predators and prey (that also is influenced by the ocean physics) this second component leads to variability in recruitment.

Fish populations are potentially able to adapt to the mean horizontal transport and dispersion pattern, i.e. the “ocean circulation climate”, by influencing the vertical position of their planktonic offspring. This occurs in two ways. Firstly, through the initial release points of the eggs that includes the spawning behavior where the fish select spawning area [2–4] and the spawning depth [5,6]. Secondly, by producing eggs of defined specific gravity [7,8] that, in turn determines the local buoyancy of the fish eggs [9,10] and hence, their vertical position in the water column [11–13]. In this way, the resulting vertical distribution of the ichthyoplankton structures the predator-prey dynamics for the pelagic offspring as well as the horizontal transport and dispersion towards the feeding areas for the young of the year. Vertical distribution of the planktonic offspring is, consequently, a key factor in understanding the ambient conditions during a period, which has strong impact on year-class formation [14–16].

Differently from invertebrates in the marine environment the teleosts have the specific attribute of maintaining approximately constant concentration of salts in their blood and body fluids [17,18] independent of the ambient seawater salinity. It implies that teleosts have active osmoregulation where salts are excreted from the body. In eggs this occurs particularly in the chloride cells embedded in the surface epithelium of the yolk sac [19]. The water content in teleost eggs is a key factor in determining egg specific gravity [20]. The eggs are keeping their internal salt concentration approximately constant by osmoregulation independent of the ambient salinity. As poikilotherms, however, the internal temperature is equal to that of the ambient seawater. This implies that the difference in specific gravity between the egg and the ambient seawater, *Δρ*, has been assumed to be largely dependent on the salinity alone (but see later in this paper the analysis on how the thermal expansion of the various chemical components of the egg influences specific gravity). The dominating role of salinity in teleost egg specific gravity has the implication that it is possible to measure rather precisely the egg specific gravity in laboratory. This was first done by embedding the eggs in salinity solutions to obtain the salinity level of neutral buoyancy [7]. Coombs (1981) [21] introduced the *density gradient column* for high-precision measurements of the specific gravity of fish eggs, which allowed for calculating vertical velocities of eggs in the water column and to develop models for their vertical distribution [11,12]. Coombs’ method became the start of a new and comprehensive understanding of the physical-biological properties of fish eggs. It became possible to observe quantitatively the impacts of the various components within the fish egg. For example, Coombs (1985) [22] addressed the impacts of thermal expansion on egg specific gravity, while the effects of egg size and chorion thickness on egg specific gravity were explored in several studies [23–27]. The development of egg specific gravity through incubation was observed [5,13,22,28,29], and the causes behind these changing patterns have been analyzed [27]. The particular impacts of buoyancy changes in eggs with large perivitelline space, like in sardine eggs, have been studied [30,31].

The first model of vertical distribution of fish eggs was developed by Sundby (1983) [11], based on the fraction of eggs with specific gravity lower than that of surface water (i.e. pelagic eggs) and a depth-independent eddy diffusivity through the mixed layer. The model was developed for steady-state distributions, which imply balance between buoyancy flux and vertical turbulent diffusion. This steady-state model concept was extended to include mesopelagic eggs and bottom eggs through the entire water column in a vertical hydrographic structure where salinity increases with depth [12]. Such vertical salinity structure is characteristic for the major parts of coastal regions of the world oceans where runoff from land and/or ice melting dominate the salinity. Coastal regions also comprise the spawning areas for the major part of marine fishes of the world oceans [32], and, hence, with an ambient hydrographic structure as described above. However, as will be shown in the present paper, in the major area of the high seas of world oceans the vertical salinity profile is opposite of most of the coastal regions, i.e. salinity decreases with depth. Also, in some specific coastal regions, particularly in the eastern boundary upwelling ecosystems (EBUEs), salinity decreases with depth. These particular ecosystems are important regions for the life cycle, including the spawning, of major fish species. EBUEs supply 20% of marine fish catches of the world oceans [33].

In this paper we expand the basic concepts of vertical distribution of marine fish eggs as earlier described for coastal regions with increasing vertical salinity with depth [11,12,34] to include ambient physical settings where the vertical salinity *decreases* with depth. We present the generic concepts of vertical distribution of fish eggs covering all types of physical (hydrographic) settings of the world oceans. Exploring the details of the physical-biological attributes of marine fish eggs from spawning throughout incubation are part of expanding these concepts. We show how decreasing salinity with depth constrains the possible depths where mesopelagic spawners can successfully reproduce, and we discuss how changes in egg specific gravity throughout incubation can contribute to keeping the eggs floating in the mesopelagic layer until hatching.

## Material and Methods

To analyze the basic principles of the influence of how salinity and temperature, respectively, influence teleost egg’s specific gravity and buoyancy we have applied observations from literature [22,24,26,27,30] on the quantitative composition of water, salt concentration, lipids and proteins in the yolk and embryo of the egg and observations on chorion volume (also consisting of proteins) and the volume of the perivitelline space (consisting mainly of seawater). Moreover, we have used values obtained from literature [24,35–40] on the specific gravities on lipids and proteins, and the algorithm for seawater density as a function of temperature and salinity [41] as shown in Table 1. We have used observations from the literature, as cited in the table legend of Table 2 [5,22,24,25,27,40,42–45], on the volumetric thermal expansion of lipids and proteins together with the volumetric thermal expansion of seawater at various salinities [41] to compare the thermal expansion of the ambient seawater and the net thermal expansion of fish eggs, as calculated according to Eq 3, and displayed in Table 2. Finally, we selected eggs from four different fish species to analyze the sensitivity of changes in thermal expansion for different compositions of perivitelline space, water content of the yolk and ambient salinity. The differences in volumetric thermal expansions are displayed in Table 2. We have used the conclusions from Table 2 - i.e. that the differences in thermal expansion of fish eggs and ambient seawater are negligible over the range of ambient salinities and species—to describe how mesopelagically spawned eggs rise or sink through the water column. Vertical velocities of the eggs were calculated according to Sundby (1983) [11] taking into account the changes in vertical speed of the eggs related to the Reynolds number. Data from the World Ocean Atlas (WOA) 2009 [46] was used to present the critical features of vertical salinity structure of the world oceans. WOA consists of objectively analyzed climatological fields, e.g. *in situ* temperature and salinity data, on a global 1°×1° longitude—latitude grid at standard depth levels. Since most mesopelagic and bathypelagic eggs are observed at various depth ranges above 500m depth four alternative depth ranges (100–500 m depth, 0–300 m depth, 100–300 m depth, and 100–400 m depth) are applied to illustrate ambient salinity structure of mesopelagically spawned eggs in general. In the regions with negative vertical salinity gradient we show that steady-state vertical distribution of mesopelagic eggs is an impossible solution. We have used observations on Cape hake (*Merluccius capensis*) eggs in the Northern Benguela to illustrate how mesopelagic eggs develop vertically under such conditions.

**Table 1:** Specific gravities, *ρ*, of the basic components in fish eggs taken from the literature. References: Liverseege (1904) [35], Huang and Sathivel (2008) [36], Yin and Sathivel (2010) [37], Navarro-Garcia *et al*. (2009) [38], Salam *et al*. (2005) [39], Tristram-Nagle *et al*. (1986) [40], Kjesbu *et al*. (1992) [24], Fofonoff and Millard Jr (1983) [41].

	Specific gravity	
Component	*ρ* (g cm^-3^)	Reference
Oil of the embryo/yolk	0.926	[35–39]
Proteins of the embryo/yolk	1.259	[40]
Chorion	1.204	[24]
Fluid of the embryo/yolk (salinity = 11) 10°C	1.00828	[41]
Perivitelline space = ambient salinity 33, 10°C	1.02539	[41]
Perivitelline space = ambient salinity 35, 10°C	1.02695	[41]

**Table 2:** Calculated volumetric thermal expansion rates, *α*_*v*_, of eggs from four different marine fish species: Sardine (*Sardina pilchardus*) off Southern England, Cape hake (*Merluccius capensis*) in the Northern Benguela, Atlantic cod (*Gadus morhua*) at the Norwegian coast, and the Baltic component of Atlantic cod (*G*. *morhua*) in the Baltic. The calculated *α*_*v*_ values are compared to that of local ambient seawater. Bold numbers show the main conclusion which is difference in *α*_*v*_between egg and the ambient seawater.

VOLUMETRIC THERMAL EXPANSION COEFFICIENTS,α_v_ (x 10^5^)	Sardine	Cape hake	Atlantic cod	Baltic cod
Estimated average α_v_ of oil ^1)^ and proteins ^2)^	65	65	65	65
*α*_v,_ yolk and embryo fluid. Range T: 8–18°C, S = 11	15.1			
*α*_v,_ yolk and embryo fluid. Range T: 10–15°C, S = 11		14.6		
*α*_v,_ yolk and embryo fluid. Range T: 5–10°C, S = 11			8.4	8.4
Calculated *α*_*v*_ for sardine eggs ^3)^. Range T: 8–18°C	20.6			
*α*_v,_ seawater of sardine eggs. Range T: 8–18°C, S = 33	19.6			
*Δα*_*v*_ between sardine eggs and the ambient seawater	**+1.0**			
Calculated *α*_*v*_ for Cape hake eggs^4)^. Range T: 10–15°C		17.6		
*α*_v,_ seawater of Cape hake eggs. Range T: 10–15°C, S = 35		19.6		
*Δα*_*v*_ between Cape hake eggs and the ambient seawater		**-2.0**		
Calculated *α*_*v*_ for Atlantic cod eggs^5)^. Range T: 5–10°C			12.1	
*α*_v,_ seawater of Atlantic cod eggs. Range T: 5–10°C, S = 34			14.2	
*Δα*_*v*_ between Atlantic cod eggs and the ambient seawater			**-2.1**	
Calculated *α*_*v*_ for Baltic cod eggs^6)^. Range T: 5–10°C				10.3
*α*_v,_ seawater of Baltic cod eggs. Range T: 5–10°C, S = 15				9.4
*Δα*_*v*_ between Baltic cod eggs and the ambient seawater				**+0.9**

The sources for the applied values:

^1)^ *α*_*v*_of marine oils is 65 x 10^−5^ (Coupland and McClements 1997) [42].

^2)^ *α*_*v*_ of proteins are ranging from 35 x 10^−5^–100 x 10^−5^ ((Tristram-Nagle *et al*. 1986 [40]; Frauenfelder *et al*. 1987 [42]; Cordier and Grzesiek 2002 [44]; Dellarole *et al*. 2013 [45]).

^3)^ Volume fractions of sardine eggs based on Coombs *et al*. (1985) [22].

^4)^ Volume fractions of Cape hake eggs based on Sundby *et al*. (2001) [5].

^5)^ Volume fractions of Atlantic cod eggs based on Jung *et al*. (2014) [27].

^6)^ Volume fractions of Baltic cod eggs based on Kjesbu *et al*. (1992) [24] and Nissling *et al*. (1994) [25].

### Ethics Statement

This paper meets all demands on ethic standards as outlined in PloS One submission guidelines.

## Results

The understanding of vertical distribution of fish eggs in the world oceans is based on the mechanism behind: 1) egg specific gravity and how it varies through incubation, 2) how egg buoyancy relates to the vertical hydrographic structures in the ocean, and 3) the causes of vertical turbulence and how it varies through the water column.

### Egg specific gravity

**Fig 1:**
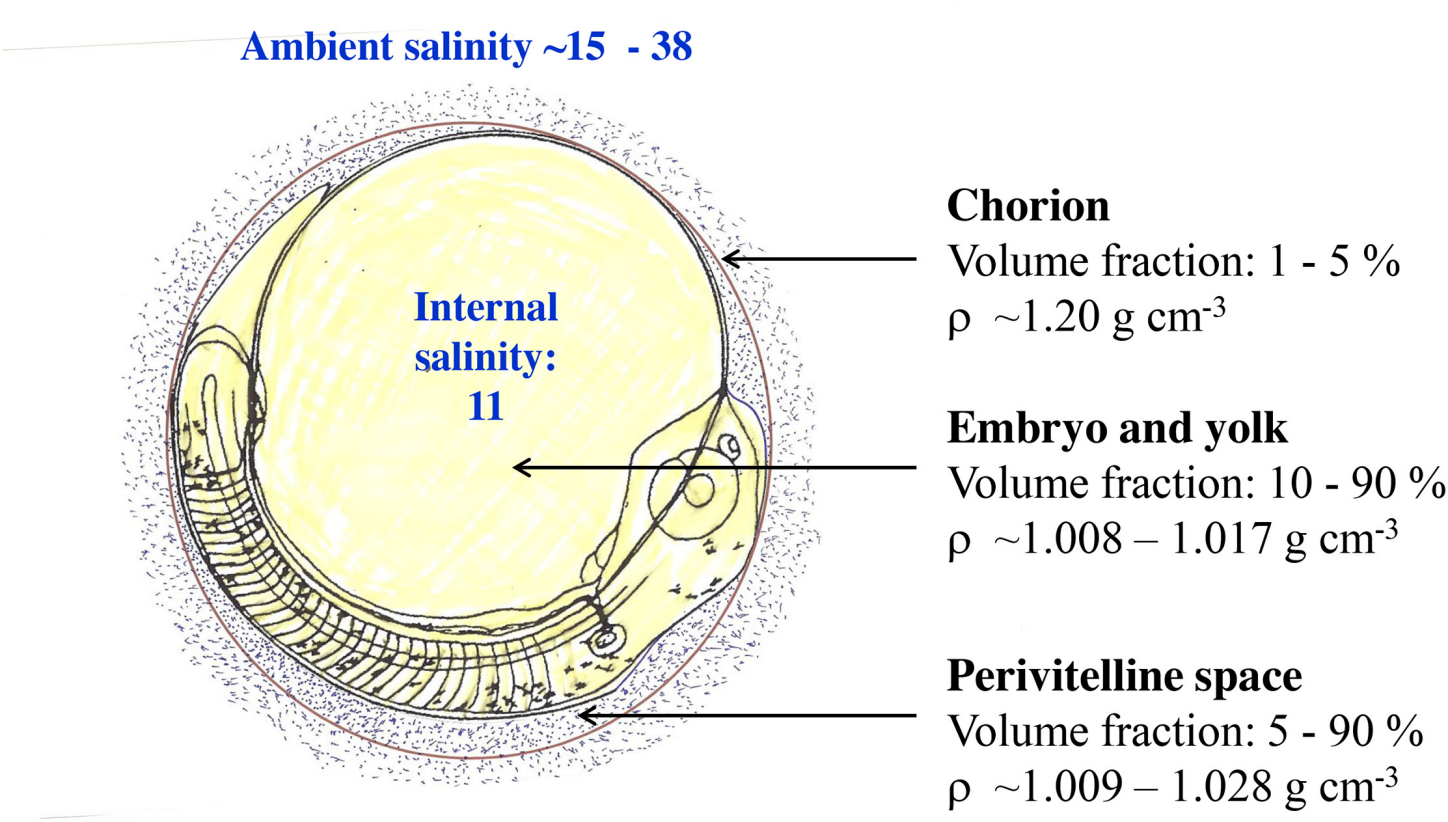
The components of teleost eggs and their physical-biological attributes. The overall specific gravity of the egg is composed of the fractional contributions of 1) chorion, 2) embryo and yolk, and 3) perivitelline space.

The mass of a fish egg consists basically of the sum of three major components (Fig 1): 1) the mass of embryo and yolk, *M*_*emb+yolk*_, surrounded by the vitelline membrane, 2) the mass of perivitelline space, *M*_periv,_ between the vitelline membrane and the chorion and 3) the chorion mass, *M*_*ch*_.

The resulting egg specific gravity, *ρ*_*egg*_, can be expressed as the fractional mass of these three components where *ρ* denotes the specific gravities and *V* denotes the volumes:

$$\rho_{egg}=\left(\rho_{emb+yolk}\cdot V_{emb+yolk}+\rho_{periv}\cdot V_{periv}+\rho_{ch}\cdot V_{ch}\right)/V_{egg}$$ (1)

The specific gravities of these three components, in turn, are determined by their composition of *lipids*, *proteins*, *water and salts*. The *lipids* and the *water* content comprise those parts of the marine fish egg that give hydraulic lift [20]. Lipids have the lowest specific gravity; typical values of fish oils are about 0.920 g cm^-3^. The specific gravity of water in fish eggs is determined by the osmotic concentration or osmolarity of the water. As mentioned above, teleosts keep the salinity of the body fluids approximately constant [17]. Riis-Vestergaard (1984) [47] measured the osmolarity of cod (*Gadus morhua*) eggs raised at ambient salinity of 34 and temperature 5°C. The average yolk osmolarity was about 320 mOsm corresponding to a salinity of 11 of isosmotic seawater. The specific gravity of seawater of salinity 11 ranges from 1.005 to 1.009 g cm^-3^ depending on the ambient temperature [41]. Consequently, when ambient salinity is higher than 11 the water content of the egg contribute to hydraulic lift, while in brackish water environment of salinity below 11 the water content of the egg is heavier compared to the ambient water and only the lipids contribute to hydraulic lift. As the micropores of the chorion allows for free flow of ambient seawater, the fluid in the perivitelline space of the egg is considered to have same salinity [48], and hence same specific gravity, as the ambient seawater. Therefore, the perivitelline space is neutrally buoyant in relation to the ambient seawater.

The heavy components of marine fish eggs are the *proteins*. These are heavier than the ambient seawater. Specific gravity of proteins varies depending on the chemical structure. The chorion material consists of the most compact proteins of the egg, but the volume of the micropores—filled with the ambient seawater—contributes to reduce specific gravity of the chorion. The average specific gravity has been measured to 1.184 g cm^-3^ for chorion in Atlantic halibut (*Hippoglossus hippoglossus*) eggs [49] and quite similar for Atlantic cod eggs, 1.204 g cm^-3^ [24]. The fractional volume of chorion, exemplified by Atlantic cod eggs, ranges from 1.5 to 4.4% and contributes to the largest single cause of intra-species variability in egg specific gravity [26]. Baltic cod, one stock under the Atlantic cod, has developed particularly large eggs with thin chorions [23] in order to be able to float high enough in the brackish water column to avoid detrimental hypoxic and anoxic conditions deeper down [50]. The volume fraction of chorion in this stock can be a low as 1.0%. However, low fractional chorion volume alone does not supply the necessary hydraulic lift of these eggs. Therefore, the proteins in the yolk of Baltic cod eggs are highly hydrolyzed resulting in the additional hydraulic lift [24,25]. Atlantic halibut (*H*. *hippoglossus*), which also has large mesopelagic eggs like the Baltic cod, have similarly thin chorion. Based on the chorion specific gravity measurement of Mangor-Jensen and Huse (1991) [49] and on the chorion mass measured by Riis-Vestergaard (1982) [51] the fractional chorion volume is here calculated to 0.9–1.0%.

We have not been able to find high-precision measurements of specific gravity of proteins in the embryo of fish eggs. However, protein material in general, in addition to bone structures, is the heaviest part of an organism. Average specific gravity of the amino acid structure in cell membrane in *Halobacterium halobium* was measure to 1.259 g cm^-3^ [40], hence somewhat heavier than the measurements on chorion [24,49].

It has been shown for a number of species that specific gravity of fish eggs all varies throughout incubation in a systematic way. Eggs from the following species have been investigated: sprat (*Sprattus sprattus*) [22], pilchard (*Sardina pilchardus*) [22], Cape hake (*M*. *capensis*) [5], blue whiting (*Micromesistius poutassou*) [13], walleye pollock (*Gadus chalcogramma*) [28], anchovy (*Engraulis encrasicolus*) [29], and Atlantic cod (*G*. *morhua*) [26,27]. The causal mechanisms behind these systematic changes were analyzed by Jung *et al*. (2014) [27]: The first stages after fertilization are characterized by increase in specific gravity due to water loss with increased salinity in the embryo and yolk [47]. The loss of water is assumed to be caused by lack of fully developed osmoregulation. Thereafter, with the increasing capability in osmoregulation, caused by the development of the embryo, the water content is restored and the egg becomes lighter. Just prior to hatching the eggs appear to become abruptly heavier while the hatched larvae, by getting rid of the heavy chorion, comes out as considerably lighter larval organism than the egg. In conclusion, the changes in specific gravity throughout incubation are mainly caused by biochemical changes within the embryo and yolk, while the chorion remains practically inert to changes. A slight reduction in chorion thickness of 1.5 to 2% towards the end of incubation, possibly linked to enzymatic breakdown of chorion linked to the hatching process, was found to have no significant influence on the overall specific gravity of the egg [27]. According to the outline above the constituents in the embryo and yolk separated in the various components of lipids, proteins, water and salt, and Eq 1 can be expressed as:

$$\rho_{egg}=\left[\left(\rho_{lipid}\cdot V_{lipid}+\rho_{protein}\cdot V_{protein}+\rho_{fluid}\cdot V_{fluid}\right)+\rho_{seaw}\cdot V_{periv}+\rho_{ch}\cdot V_{ch}\right]/V_{egg}$$ (2)

where *ρ*_*lipid*_ and *V*_*lipid*_ are the specific gravity and volume, respectively, of lipids within the embryo and yolk, *ρ*_*protein*_ and *V*_*protein*_ are the specific gravity and volume, respectively, of the proteins within the embryo and yolk, and *ρ*_*fluid*_ and *V*_*fluid*_ are the specific gravity and volume, respectively, of the fluid content occupied by the embryo and yolk. From Eq 2 it is possible to analyze the relative effects of the various components on egg specific gravity from a general approach independent of species. Table 1 shows the specific gravities, *ρ*, of the basic components in fish eggs. From the literature describing a number of fish species from various parts of the organisms (i.e. liver, tail, brain, ventral, dorsal fin) the oil shows small variation, ranging from 0.919 to 0.939 g cm^-3^ and with the mean value of 0.926 g cm^-3^ (see the publications listed in the table legend). There was no apparent temperature trend for the observations (between 15 and 25°C) and no apparent trends neither between species nor organs. The only observation of oil in fish eggs was from the fish species Hilsa (*Tenualosa ilisha*) (Hamilton, 1822) [39] and was 0.926 g cm^-3^, identical to the above mean value. We interpret these results that oils in marine fish show quite similar values of specific gravity among organs. For proteins Tristram-Nagle *et al*. (1986) [40] observed mean specific gravity of amino acids to 1.259 g cm^-3^, while the chorion in Atlantic cod (*G*. *morhua*), which consists of hardened proteins, was somewhat lighter 1.204 g cm^-3^ [24]. The difference could be explained by the fact that the micropores of the chorion are occupied by the lighter seawater. The saline water components of the eggs can be calculated at very high precision [41], including the internal fluids of the embryo and yolk and the seawater of the perivitelline space.

### Egg buoyancy

The major fractions of marine fish eggs are pelagic or mesopelagic implying that they float above the seabed. This is different from fish eggs in freshwater environments where eggs are found benthic, a natural consequence of negative buoyancy of the egg’s water content in freshwater because of the osmolarity level in fish eggs, as outlined above. Hence, only the lipids in the fish eggs can contribute to hydraulic lift in freshwater; both the proteins and the fluids will make the fish egg sink. Georg O. Sars [52] was the first to observe that marine fish eggs could float when he sighted cod eggs in large quantities at the surface and by sampling in plankton net at the spawning areas of Barents Sea (Northeast Arctic) cod in Lofoten, northern Norway. This means that the eggs are positively buoyant compared to the specific gravity of the surface waters: *Δρ* = *ρ*_*w*_ –*ρ*_*egg*_ > 0, where *ρ*_*w*_ is the specific gravity of the surface water, and *Δρ*_*egg*_ is the egg specific gravity. Typical buoyancy values for pelagic eggs are less than 0.006 g cm^-3^ [11] with the major part of them ranging between 0.001 and 0.002 g cm^-3^ (i. e. in hydrography terms between 1 and 2 σ_t_ units). As outlined in the Introduction, the salinity is the determinant factor for the egg buoyancy, *Δρ* [7] as fish eggs keep their internal salinity nearly constant independent of ambient salinity, while the temperature does not deviate from the ambient temperature. However, this concept is based on the assumption that the thermal expansion of the fish egg is equal to that of the ambient seawater. Although the major part of the egg consists of water this cannot be exactly true, since the fractions of the egg consisting of lipids and proteins have larger thermal expansions than that of seawater. According to Coombs *et al*. (1985) [22] this was already stated by Remotti (1921) [53]. Coombs *et al*. (1985) [22] explored this issue in more detail on sprat (*S*. *sprattus*) eggs and pilchard (i.e. sardine) (*S*. *pilchardus*) eggs by observing changes in specific gravity in the density gradient column [21] under oscillating temperatures ranging from 6 to 12°C for sprat and from 8 to 18°C for pilchard. Surprisingly, the observed thermal expansion for the eggs was not higher but slightly lower than for the ambient seawater of salinity 33: The mean volumetric thermal expansion, *α*_*v*_, of seawater of salinity 33 in the range 8–18°C is 19.6 ×10^−5^ /°C, while the observed *α*_*v*_ for the pilchard eggs in the same temperature range was observed to be 18 ×10^−5^. Coombs *et al*. (1990) [54] made similar experiments on plaice eggs *(Pleuronectes platessa*) from the southern North Sea and found that the thermal expansion rate of local seawater was 15.3 ×10^−5^ /°C compared to 14.0 ×10^−5^ /°C for the plaice eggs, and hence a similarly lower expansion rate of the pilchard eggs and plaice eggs.

The explanation to the results of Coombs *et al*. (1985; 1990) [22,54] could not be because the contribution of thermal expansion from lipids and proteins are negligible compared to the thermal expansion of the fluids in the fish egg. To compare with the observations of Coombs *et al*. (1985) [22] we have calculated the volumetric thermal expansion coefficients, *α*_*v*_, for sardine eggs in Table 2 according to:

$$\alpha_{v\;egg}=\left[\left(\alpha_{v\;oil\;and\;proteins}\right)\cdot \left(V_{oil\;and\;proteins}\right)+\left(\alpha_{v\;sal=11}\right)\cdot \left(V_{yolk\;and\;embryo\;fluids}\right)+\left(\alpha_{ambient\;sal}\right)\cdot \left(V_{periv}\right)\right]/V_{egg}$$ (3)

For comparison and assessment of the robustness of the calculations we have also calculated similar values for Cape hake eggs in the Northern Benguela, Atlantic cod eggs at the Norwegian Coast, and Baltic cod eggs. The calculations are based on *α*_*v*_ for the basic components oil and proteins taken from the literature as cited in Table 2. From the table it is evident that both components have considerably higher thermal expansion rates than saline water. Marine oils have a volumetric thermal expansion coefficient, *α*_*v*_, of about 65 ×10^−5^ / °C [42], This is 3.5 times the *α*_*v*_ of the pilchard egg or seawater of salinity 33. The *α*_*v*_ of proteins varies somewhat depending on structure of the protein, from 35 × 10^−5^ to 100 × 10^−5^ / °C [40,43,44,45], i.e. from about 2–5 times the volumetric expansion of the pilchard egg or the seawater. Existing literature only give us data on total sum of dry weights and water, and not data on fractional contribution on lipids and proteins except for some single species as assembled by Riis-Vestergaard (2002) [18]. Therefore, we have here assumed similar values of *α*_*v*_ for the lipids and proteins of the eggs in the calculations. For the present purpose the assumption should be justified because the average volumetric thermal expansion rate of the proteins is quite similar to the value of oil, i.e. 67 × 10^−5^ compared to 65 × 10^−5^ / °C. For seawater the *α*_*v*_ decreases with decreasing salinity as well as decreasing temperature [41]. This implies that the water content of the yolk and embryo (with approximate salinity of 11) has lower *α*_*v*_ than that of the ambient seawater, as appears from Table 2. The *α*_*v*_ of the perivitelline space is identical to that of the ambient seawater as the salinity is identical across the chorion. Hence, the 3.5 times larger *α*_*v*_ of proteins and oil in the yolk and embryo than that of the ambient seawater is counterbalanced by the lower *α*_*v*_ of the water (of salinity ~11) inside the vitelline membrane. Thermal expansion in seawater increases with salinity. For example, the *α*_*v*_ of the water content inside the vitelline membrane is 0.71 times that for seawater of salinity 35. Apparently—by chance—this results in quite small differences in *α*_*v*_ between the fish egg as a whole and the ambient seawater (shaded values in Table 2) with the variations between species depending on the ambient salinity and the volume fractions of the basic components of the egg (Eq 3). This is also confirmed by the measurements of sardine eggs [22]. Sardine eggs deviate from most marine fish eggs by having extraordinary large perivitelline space, up to 90% of the total egg volume and, hence, the larger part of the sardine egg has identical *α*_*v*_ to that of the ambient water. In contrast, the perivitelline space in cod eggs is about 10% of the total egg volume. The difference in *α*_*v*_ between the cod egg and the ambient seawater is still small, 2.1 × 10^−5^ / °C, but with opposite sign, because the larger volume fraction of water in the yolk and embryo in cod eggs contributes to less thermal expansion of the eggs in total than of the ambient seawater. The same trend is apparent for Cape hake eggs in the Northern Benguela. However, similarly to sardine eggs, Baltic cod eggs show slightly larger expansion than the ambient seawater. This can be explained by the fact that the ambient seawater of Baltic cod eggs has a low salinity of 15–16 and hence only slightly higher than the internal salinity of the yolk and embryo. This results in small difference in *α*_*v*_ of the internal fluid and ambient seawater.

In conclusion, the thermal expansion of fish eggs, in general, and the ambient seawater are not equal, but the differences are so small that they are difficult to measure. Potentially, it is only for fish eggs rising through a water column of large temperature changes and where egg buoyancy, *Δρ*, is small that differences in thermal expansion could have an effect on the rising speed of the eggs. Such a situation could be for Cape hake eggs rising from around 400 m depth in the Northern Benguela as described by Sundby *et al*. (2001) [5]. Here, the hake eggs rise during incubation from spawning at about 10°C at 400 m depth to nearly 15°C in the subsurface layers at hatching. The mean buoyancy, *Δρ*, of the eggs is 0.0011 g cm^-3^. A lower thermal expansion rate for the hake eggs of 2 × 10^−5^ /°C relative to the ambient seawater (according to Table 2) would then reduce the buoyancy from 0.0011 to 0.0010 g cm^-3^ by the end of ascent when they reach the 5°C warmer surface layers. This would, in turn, reduce the ascending speed of the average size hake egg (i.e. 0.95 mm in diameter) from 0.41mm s^-1^ at 400 m depth to 0.39 mm s^-1^ at the subsurface layers. The relatively lesser reduction in ascending speed is because of lower viscosity at higher temperature that causes higher ascending speed.

To summarize the present paragraph on egg buoyancy: the *in-situ* specific gravity of a fish egg, *ρ*_*eggD*_, can be expressed as a function of the *in-situ* temperature, T_D_, at the depth *D* where the egg is located and on the salinity of neutral buoyancy measured in the laboratory, *S*_*L*_. Hence, *ρ*_*eggD*_ = *f (T*_*D*_, *S*_*L*_ *)*. The function, *f*, (as displayed in Fofonoff and Millard (1983) [41]) shows increasing specific gravity, *ρ*, with decreasing temperature, *T*, and increasing *ρ*, with increasing salinity, *S*. The actual specific gravity of the ambient water, *ρ*_wD,_ for the egg is a function of *T*_*D*_ and the salinity at depth, *S*_*D*_: *ρ*_*wD*_ = *f (T*_*D*_, *S*_*D*_ *)*

Whether the egg will rise or sink at the local, *in-situ*, depth, *D*, is therefore only dependent on difference in salinity of egg neutral buoyancy (measured in the laboratory), *S*_*L*_, and salinity at the *in-situ* depth, *S*_*D*_. Consequently, if *S*_*L*_ *< S*_*D*_, the egg will rise at the local, *in-situ*, depth; if *S*_*L*_ *> S*_*D*_ the egg will sink.

The calculations across the four different species under a variety of ambient hydrographic conditions demonstrate the robustness of this conclusion, and the insensitivity of the *in-situ* temperature on egg buoyancy will be used in the subsequent consideration on vertical distributions of fish eggs of the deep sea.

### Steady-state vertical distributions of fish eggs

**Fig 2:**
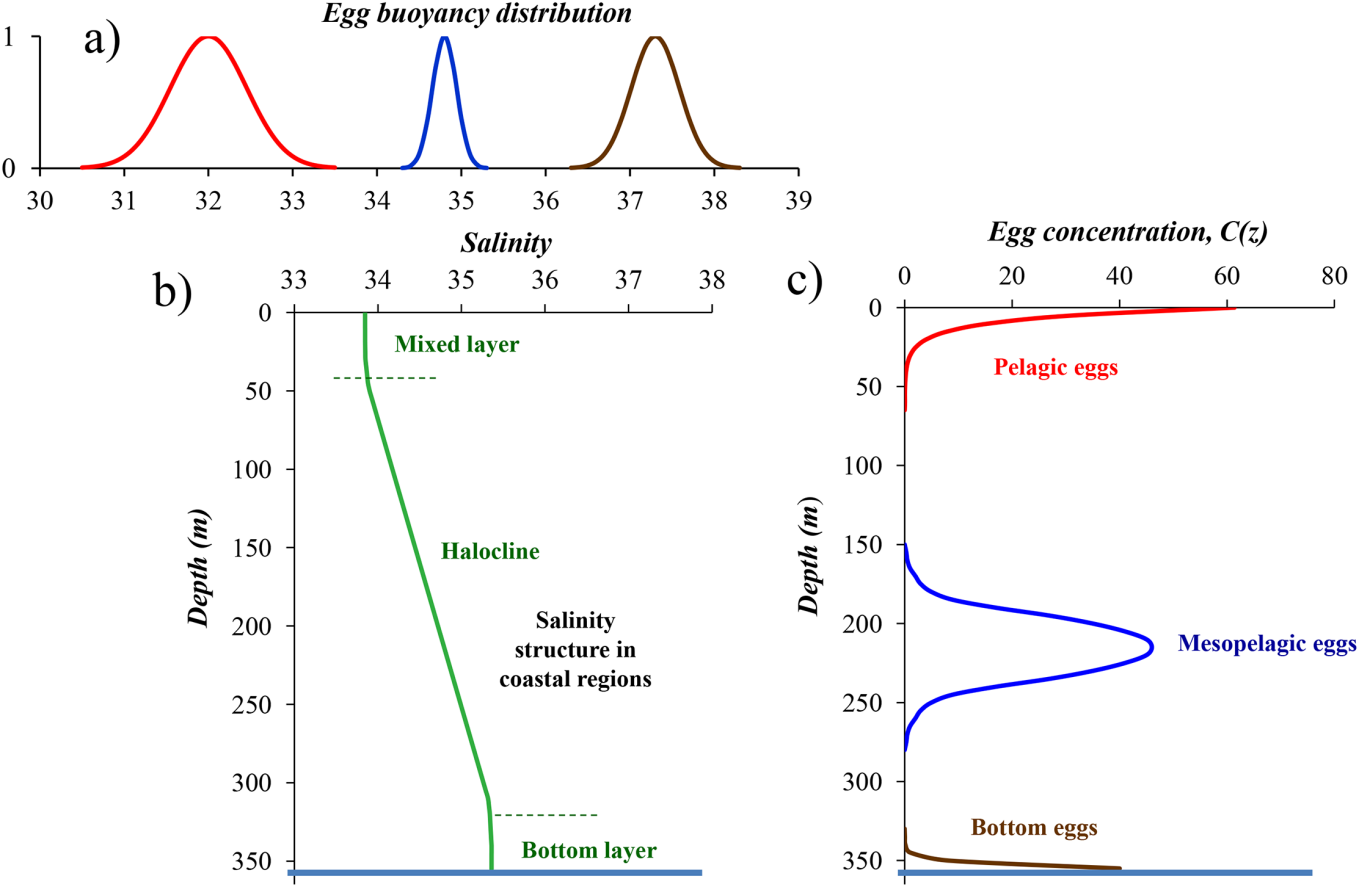
Principles of three main types of specific gravities of eggs and the resulting vertical distributions. a) egg buoyancy distribution of the three major types of marine fish eggs. Red: pelagic eggs. Blue: mesopelagic eggs. Green: bottom eggs, b) vertical salinity structure in coastal regions, c) vertical distribution of the three major types of marine fish eggs. After Sundby (1997) [34].

A major part of marine fish eggs are confined to the upper mixed layer, and are termed *pelagic eggs*. The physical dynamics of such vertical distributions were outlined in Sundby (1983) [11] who developed a vertical model for the distribution of pelagic eggs. Pelagic eggs have positive buoyancy in relation to the upper mixed layer implying that the buoyancy, *Δρ* = *ρ*_*w*_ –*ρ*_*egg*_, (where *ρ*_*w*_ is the specific gravity of the mixed layer and *ρ*_*egg*_ is the specific gravity of the egg) has positive value giving the eggs a hydraulic upward lift. This hydraulic lift, or the buoyancy force, *w* · *C(z)*, (where w is the vertical ascending speed of the egg and *C(z)* is the change in egg concentration with the depth, *z*) is balanced by the vertical mixing, *K* · *dC(z)*/*dz*, under steady-state conditions. The eddy diffusivity coefficient, *K*, is large and strongly variable in time in the upper mixed layer mainly due to the varying action of wind mixing, but also in certain areas due to strong tidal mixing. The solution to the balance between these two forces under steady-state conditions is:

$$C\left(z\right)=C\left(a\right)\cdot e^{-\left(\frac{w}{K}\right)\cdot \left(z-a\right)}$$ (4)

where *C(a)* is the egg concentration at a given depth *z* = *a*. The profile is illustrated in Fig 2. Eq 4 simply shows that the vertical egg profile is determined by the ratio between ascending velocity, *w*, and the eddy diffusivity coefficient, *K*. This coefficient varies by more than one order of magnitude induced by the variable winds while the natural span of buoyancy in pelagic eggs, *Δρ* is typically varying from 0.001 to 0.003 g cm^-3^. From Eq 4 it is evident that changes in *K* are contributing much more to changes in the ratio *w*/*K*, and therefore also to the vertical distribution of pelagic eggs compared to variations in egg buoyancy. Observations of such distributions were done by Iversen (1973) [55] who observed the vertical distributions of Atlantic mackerel (*Scomber scombrus*) eggs in the North Sea by Clark-Bumpus plankton samplers under various weather conditions from 0 to 12 ms^-1^ of wind speed. Similarly, measurements of vertical distribution of Atlantic cod eggs were conducted by egg pump in Lofoten, Northern Norway, over a range of wind speeds [56].

The above model has been further developed by several authors to account for density stratification in the upper layer and for depth-dependent eddy diffusivity coefficient, *K*(*z*), and for non-stationary conditions [30,57–59]. For example, an important modification of the original simplified model is that depth-dependent *K*(*z*) in combination with a homogeneous mixed layer can results in displacement of the maximum egg concentration from the surface to levels deeper down in the mixed layer [60,61], which was also confirmed by their observations.

Eggs that have higher specific gravity compared to the upper mixed layer water will sink out until they reach the neutral buoyancy level. In coastal waters, where most of the world oceans’ fish species spawn [32], the salinity increases with depth and the majority of fish eggs with higher specific gravity than that of the upper mixed layer will float in the halocline at the salinity level corresponding to neutral buoyancy [34]. Examples of such mesopelagic egg distributions are coastal cod (*Gadus ogac*) eggs in West Greenland fjords [62], Atlantic halibut (*Hippoglossus hippoglossus*) eggs in Norwegian fjords [63,64], and walleye pollock (*Gadus chalcogramma*) eggs in the Shelikof Strait, Gulf of Alaska [65]. A number of fish species in the Baltic Sea, where the upper brackish water layer has too low specific gravity for eggs to float, have mesopelagic egg distributions such as cod (*G*. *morhua*) [23,66], flounder (*Platichthys flesus*), and plaice (*Pleuronectes platessa*) [66]. In regions with higher surface salinities, such as in the North Sea, these species would float as pelagic eggs, for example the North Sea plaice eggs [11,67]. As for the Baltic Sea, mesopelagic fish egg distributions also seem to dominate in the Black Sea, particularly the Atlantic bluefin tuna (*Thunnus thynnus*) eggs [68].

While the vertical velocity of pelagic eggs were considered constant through the mixed layer (*w*(*z*) = constant) in the development of Eq 4 the vertical velocity of mesopelagic eggs in the halocline will vary with the distance from the level of neutral buoyancy. Under the assumption of a linear halocline the vertical speed of the fish egg can be expressed as a linear function of depth *w*(*z*) = *k* · *z* + *b* where *k* describes the slope of the density gradient. Following the same reasoning of balance between the buoyancy forces and vertical diffusive flux as for the development the vertical distribution of pelagic eggs (Eq 4), the vertical distribution of mesopelagic eggs in a linear pycnocline can be expressed according to Sundby (1991) [12] and similar to the normal distribution function (Fig 2):

$$C\left(z\right)=C_{A}\cdot e^{-\left(\frac{m}{2K}\right)\cdot {\left(z-z_{A}\right)}^{2}}$$ (5)

where *C*_*A*_ is the egg concentration at the depth level *z*_*A*_ where the egg neutral buoyancy is found and m is a constant proportional to the density stratification, and hence also proportional to the vertical speed of the eggs, *w*(*z*), since *w*(*z*) is linearly proportional to *Δρ* in the Stokes equation (See Sundby (1991) [12] for details). Differently from the mixed layer, the eddy diffusivity coefficient, *K*, is very small in the subsurface pycnocline, typically 4 to 5 orders of magnitude lower [34]. Therefore, the variation in buoyancy distribution of the eggs, *Δρ*, determines the vertical distribution of mesopelagic eggs. The variation in *K* is negligible for the changes in vertical position of the eggs, and hence opposite of the pelagic eggs.

For some species in certain regions the heaviest fraction of the eggs are mesopelagically distributed while the lightest fraction is pelagically distributed, such as eggs from anchovy (*E*. *encrasicolus*) and sardine (*S*. *pilchardus*) in the near-coastal regions with lower salinity in the Bay of Biscay [30,31], cod (*G*. *morhua*) eggs in the Northern Gulf of St. Lawrence [69,70], and coastal cod (*G*. *morhua*) eggs in Norwegian fjords [71–73]. For increasing salinity with depth (Fig 2) eggs will float when they have neutral buoyancy, expressed in salinity, S_L_, lower than the salinity at the bottom.

Most fish eggs in freshwater are benthic while such distributions are rare in the marine environment, as outlined above. However, some few marine species have benthic eggs, for example herring (*Clupea harengus*). The herring eggs are, however, sticky and they are spawned on high-energy bottom habitats attached to vegetation or gravel or rocky substrates [74]. Benthic eggs that are spawned on the bottom without being attached to the bottom substrate or each other are rare, but Pacific cod (*Gadus macrocephalus*) eggs are only slightly adhesive and lose their adhesiveness throughout development [75]. Capelin (*Mallotus villosus*) eggs spawned at the coast of Finnmark in the Barents Sea seem to have similar physical properties and are mixed up in the water column from wave action [76]. The vertical distribution of such kinds of benthic eggs could be modeled similarly to that of pelagic eggs:

$$C\left(z\right)=C\left(a\right)\cdot e^{\left(\frac{w}{K}\right)\cdot \left(z-a\right)}$$ (6)

where the egg concentration decreases exponentially from seabed (Fig 2), i.e. inversely compared to the pelagic eggs.

### Buoyancy under decreasing salinity with depth in the world oceans

**Fig 3:**
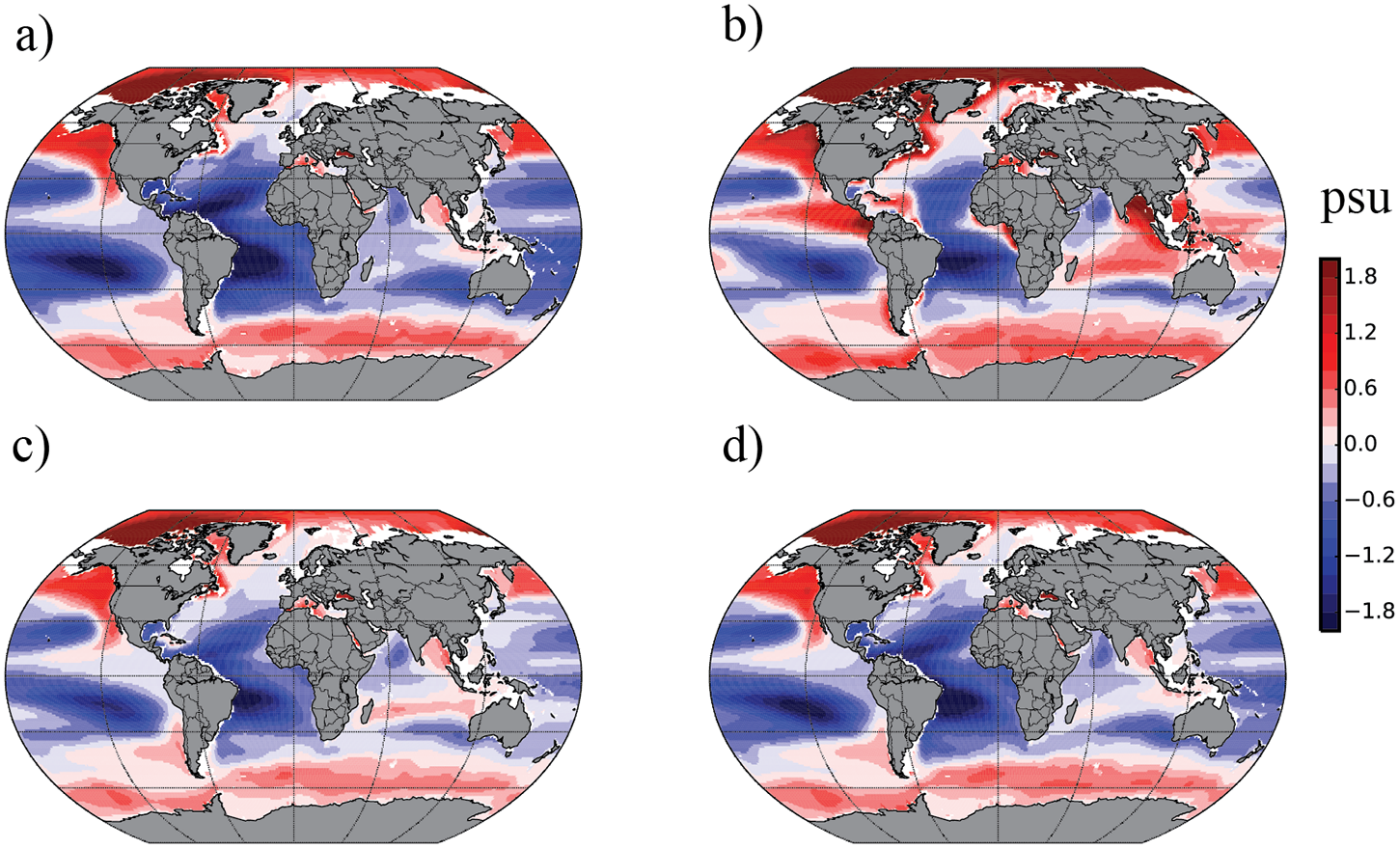
Differences in vertical salinity (*ΔS*) of the world’s oceans in four depth intervals. Salinity increases with depth in the red regions. Salinity decreases with depth in the blue regions. a) Salinity difference between 100 m and 500 m depth, *ΔS* = *S*_*100*_ *-S*_*500*_. b) *ΔS* = *S*_*0*_ *-S*_*300*_. c) *ΔS* = *S*_*100*_ *-S*_*300*_. d) *ΔS* = *S*_*100*_ *-S*_*400*_.

The steady-state vertical egg distributions outlined above with the three main types of distributions are based on a vertical salinity structure of the water column as shown in Fig 2, i.e. increasing salinity with increasing depth. Although this is the situation for the major part of spawning areas of marine fish, the major regions of the open ocean have an opposite vertical salinity structure, i.e. salinity decreases with depth. Also in certain parts of coastal regions, like in the fish-rich eastern boundary upwelling ecosystems (EBUEs) of Benguela, the Canary, and in the Chile-Peru, salinity decreases with depth. The blue areas in Fig 3 show the ocean regions where salinity decreases with depth. Steady-state distributions of pelagic eggs are also abundant in these ecosystems, just like in those parts of the world oceans where salinity increases with depth, and is shown in a number of studies from various sea regions. The small pelagic fish like anchovies, sardines and sardinella are all abundant species in EBUEs where they all have pelagic egg distributions.

Olivar (1990) [77] measured the vertical egg distribution of a number of species in the Northern Benguela that appeared to have a pelagic distribution, i.e. horse mackerel (*Trachurus capensis*), anchovy (*E*. *capensis*), sardine (*Sardinops ocellatus*) and the blenniid (*Parablennius pilicornus*). Stenevik *et al*. (2001) [78] measured the buoyancy, as well as the vertical distribution, of sardines (*Sardinops sagax*) in the Northern Benguela and confirmed that they had positive buoyancy and were pelagically distributed. Parada *et al*. (2003) [79] observed and modeled the vertical distribution of anchovy (*E*. *capensis*) in the Southern Benguela. Also in the Californian upwelling ecosystem sardine (*Sardinops caeruela*) and anchovy (*Engraulis mordax*) are found as pelagic eggs [80], although this upwelling ecosystems differs from the Benguela, Canary and Chile-Peru that in large parts of the California Current salinity increases with depth.

There is one basic difference between fish egg distributions under *decreasing* salinity with depth compared to *increasing* salinity. Under the latter conditions all kinds of fish eggs will reach a vertical equilibrium distribution independent of at which depth the fish egg is spawned—given that the eggs do not hatch before they reach equilibrium distribution. The eggs will always reach either a steady-state pelagic distribution, a mesopelagic distribution, a bottom distribution (as described in Eqs 4, 5 and 6) or a combination of these three kinds of distributions depending on the egg buoyancy distribution. This is not the case in areas of decreasing salinity with depth as displayed in Fig 3. However, also in these regions the water column itself is stably stratified, because the decreasing temperature contributes more to increase the specific gravity than the decreasing salinity contributes to decrease it. However, the egg specific gravity at a certain depth D, *ρ*_*eggD*_, is linked to the *in-situ* temperature, *T*_*D*_, but to the measured neutral buoyancy salinity from the laboratory, *S*_*L*_, On the other hand, the local specific gravity of the ambient water, *ρ*_*wD*_, is linked to the *in-situ* temperature, *T*_*D*_, and the *in-situ* salinity, *S*_*D*_. Hence, the *in-situ* egg buoyancy is *Δρ = ρ*_*wD*_ *-ρ*_*eggD*_, and the egg will rise if it is positive and sink if it is negative. As egg temperature is equal to *in-situ* temperature, T_D_, positive or negative *in-situ* egg buoyancy, *Δρ*, is determined by *ΔS = S*_*D*_ *—S*_*L*_

If the buoyancy of the egg, in terms of salinity (*ΔS = S*_*D*_ *—S*_*L*_), is less than the salinity difference between the surface and bottom (*S*_*0*_ *—S*_*B*_), *the depth at which the eggs are spawned determines whether the eggs will be able to rise towards the surface* and reach pelagic egg distribution. This implies that the surface egg buoyancy in terms of salinity difference, *ΔS*, between the egg neutral buoyancy (in salinity terms), *S*_*0*_ *—S*_*L*_, must be greater than salinity difference between the sea surface salinity, *S*_*0*_, and the salinity at the depth where the eggs are spawned, *S*_*sp*.*depth*_:

$$S_{0}-S_{L}>\;S_{0}-S_{sp.depth}\;\;\;implying\;that\;\;\;S_{L}<S_{sp.depth}$$ (7)

**Fig 4:**
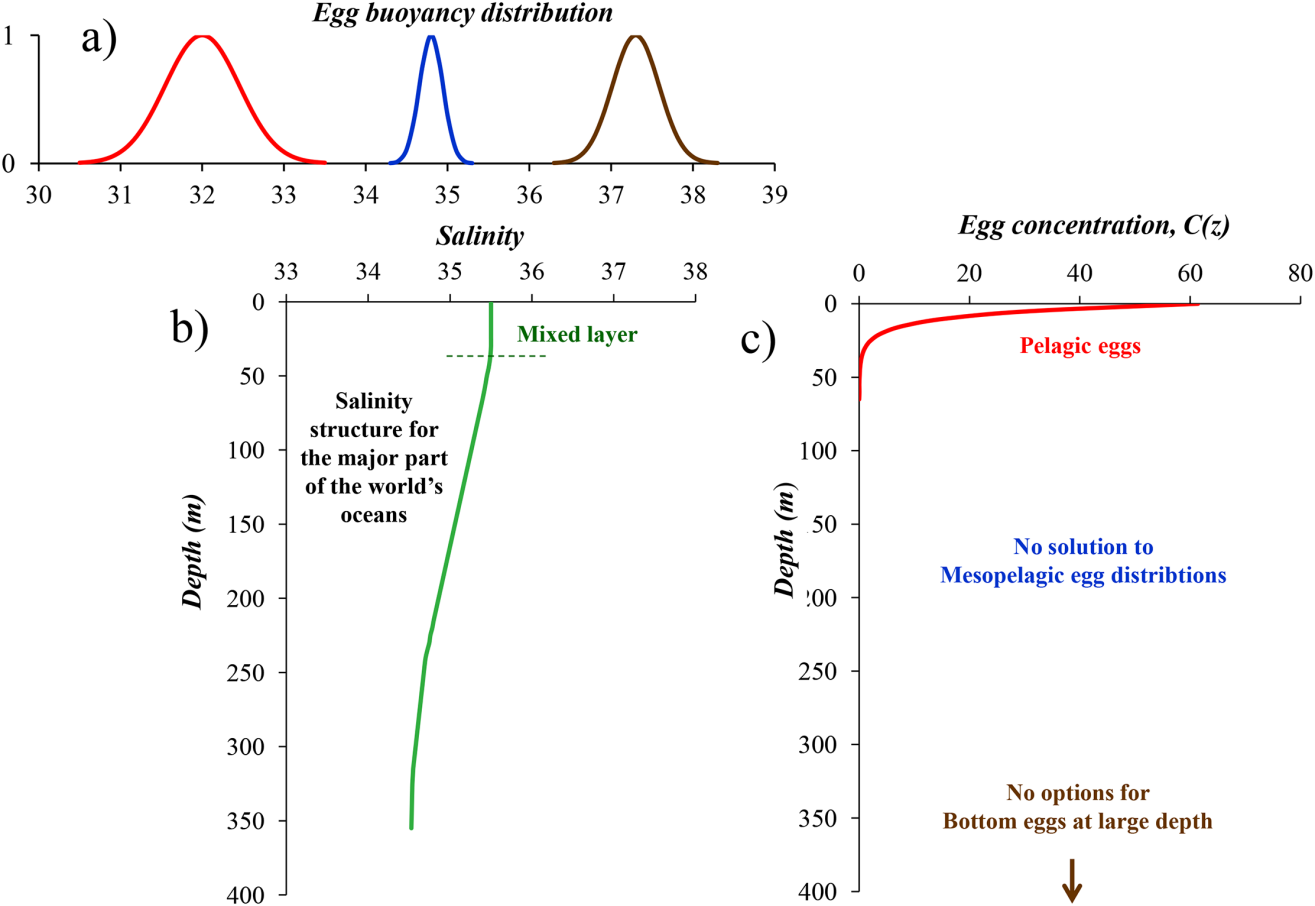
Principles of specific gravities and vertical distributions of eggs under decreasing salinity with depth. a) egg buoyancy distribution of the three major types of marine fish eggs, b) vertical salinity structure in oceanic regions, c) vertical distribution of pelagic fish eggs.

Unless this condition is met, the eggs will sink out of the water column. In most regions of the world oceans where salinity decreases with depth (Fig 3) this implies that the eggs will sink out to bottom depths of several thousands of meters where the hatched eggs will not meet surviving conditions to recruit to the stock. Fig 4 illustrates the principles of egg buoyancy and egg distribution under decreasing salinity with depth. It implies that only the pelagic egg distribution is a possible solution to vertical equilibrium distribution of fish eggs, with the additional requirement that the relation in Eq 7 is fulfilled. Steady-state mesopelagic egg distributions (Eq 5) cannot exist, such as for Atlantic halibut (*H*. *hippoglossus*) in Norwegian fjords [63,64], walleye pollock (*G*. *chalcogramma*) in the Shelikof Strait, Gulf of Alaska [65], coastal cod (*Gadus ogac*) in West Greenland fjords [62], Atlantic bluefin tuna (*T*. *thynnus*) in the Black Sea[68], and Baltic cod (*G*. *morhua*) [23,66] Bottom egg distributions (Eq 6) on continental shelves, such as for Pacific cod (*G*. *macrocephalus*) in the Shelikoff Strait [75] and capelin (*M*. *villosus*) in the Barents [76], will not be an alternative solution for surviving conditions for the larval offspring since bottom depth in these regions are typically 1000 m and deeper, and near the coast in the EBUEs hypoxic to anoxic conditions prevail near the bottom [33]. In conclusion, we therefore define a *critical spawning depth layer* for eggs with neutral buoyancy corresponding to a salinity higher than that of the deep layer. Spawning must take place shallower than the critical layer in order to prevent the eggs to sink out of the water column.

As outlined above eggs from small pelagic species, for example sardines and anchovies, are typical for the EBUEs where salinity decreases with depth. Spawning from these species occurs normally in the surface layers of the ocean, consequently meeting the demand of Eq 7. Their pelagic distribution and the shallow spawning are also the reason why such eggs are well suited for egg surveys by Continuous Underway Fish Egg Sampler (CUFES) [81]. Other kinds of vertical fish egg distributions do, however, exist also in the world’s oceans where salinity decreases with depth, but such eggs cannot have stable, equilibrium distributions. They must either be on continuous ascent or decent (Fig 4) implying that their vertical positions, as for example observed in plankton nets, are stage-dependent. Examples of such egg distributions are Cape hake (*M*. *capensis*) eggs in the Northern Benguela upwelling ecosystem [5]. It was shown that Cape hake in this region has developed a particular spawning behavior to bring the offspring inshore to the plankton-rich first-feeding areas. Spawning occurs in the deep layers (~200–350 m depth) in the offshore regions. The eggs are only slightly positively buoyant, and rise slowly towards the surface layers. As they rise they are carried onshore by the subsurface onshore compensation current below the offshore-directed Ekman layer. The ascending speed is so low that most of the eggs hatch before they reach up into the offshore-directed transport of the Ekman layer at about 25 m depth. In conclusion, the hake offspring is retained inshore, and increased upwelling is increasingly retaining the offspring inshore. Hence, increased upwelling has the opposite effect on Cape hake larval retention in the Northern Benguela compared to small pelagic fish in the Canary Current as described by Cury and Roy (1989) [82] that lead to the “Optimal Environmental Window” recruitment hypothesis.

**Fig 5:**
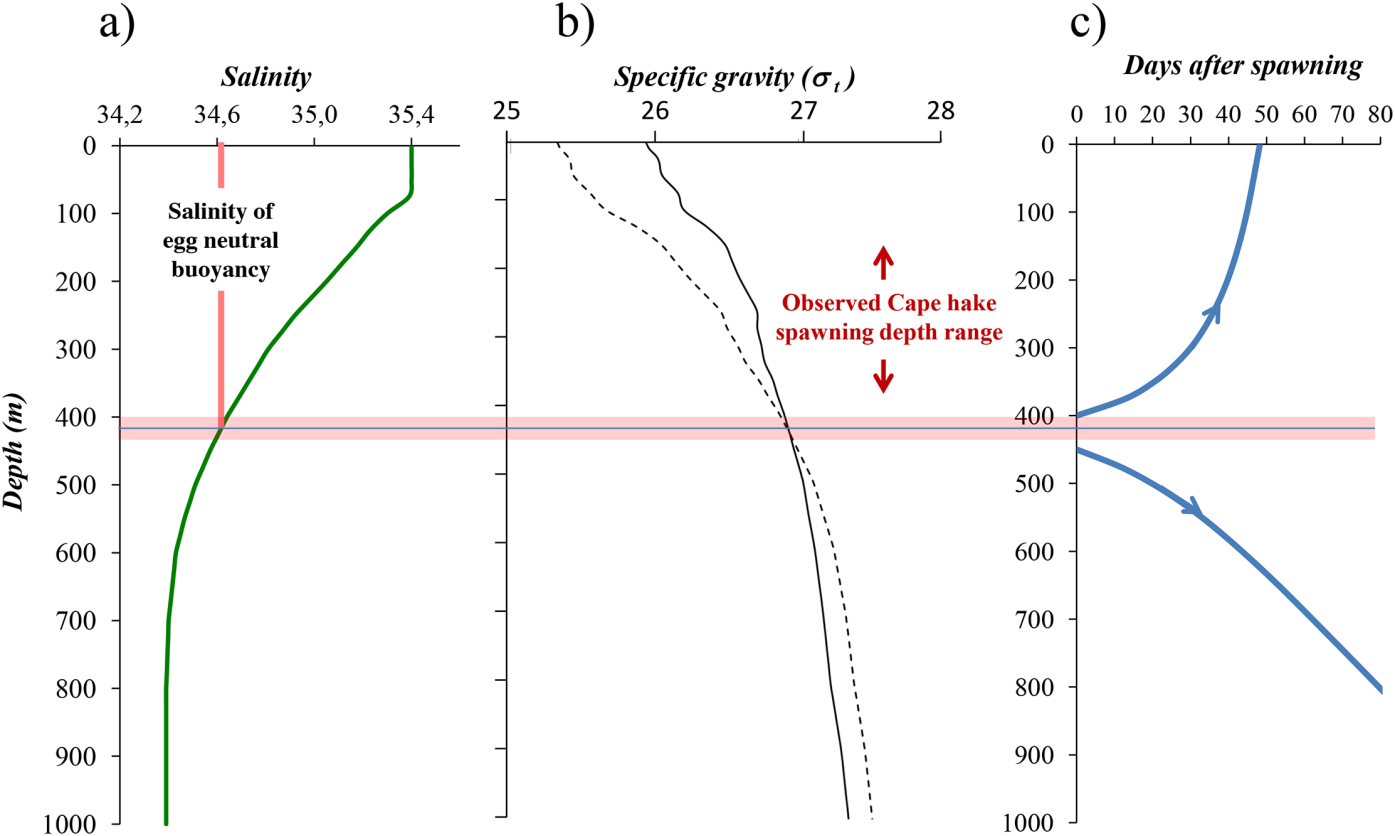
Hake spawning and egg distributions in the Northern Benguela Current. a) yearly mean salinity profile in the central Northern Benguela upwelling ecosystem at the main spawning areas of Cape hake and the mean egg neutral buoyancy. b) specific gravity in units of σ_t,_ (ρ = 1 + σ_t_×10^−3^) of the water column (whole line) and the Cape hake egg with with neutral buoyancy at salinity of 34.6 (dashed line)c) modeled ascending and descending of eggs (of neutral buoyancy at salinity of 34.6) spawned at 400 and 450 m depths, respectively. The critical spawning depth is at 425 m. Critical spawning depth layer for the population ranges between 400 and 450 m depth.

Fig 5a shows the yearly mean salinity profile taken from World Ocean Atlas from National Oceanographic Data Center [83] in the central Cape hake spawning areas of the Northern Benguela upwelling ecosystem. The mean neutral buoyancy (expressed in salinity) of Cape hake eggs, as taken from Sundby *et al*. (2001) [5], is indicated in the left panel implying that the neutral egg buoyancy in terms of salinity is found at 425 m depth. Fig 5b shows the resulting profiles of specific gravity of the water column (whole line) and the *in-situ* egg specific gravity (dashed line) based on the temperature and salinity from the World Ocean Atlas [83]. Based on these results Fig 5c shows the calculated accumulated ascending and descending of Cape hake eggs spawned at 400 and 450 m depth, respectively. The initial speeds are very small as the buoyancy, *Δρ*, is at the minimum. The speeds of the eggs increase as their vertical levels changes and the positive/negative buoyancies of the eggs increase.

Although the exact critical spawning depth for a hake egg of salinity neutral buoyancy equal to 34.6 is at 425 m depth, it is more relevant to define a critical spawning depth layer as shown in Fig 5, because a fish population does not produce fish eggs with exact equal buoyancy. Moreover, internal breaking waves may result in eggs spawned above the critical spawning depth to be mixed down below and thereafter sink out of the water column. Therefore, the critical spawning depth layer of the Cape hake population in the Northern Benguela is here defined as the depth layer from 400 to 450 m depth as shown in Fig 5. The actual observed spawning depths are from around 200 to 350 m depth. Hence, Northern Benguela Cape hake appears to have adapted to the critical spawning depth layer.

Another documented example of deep spawning in a situation of decreasing salinity with depth is the blue whiting spawning to the west of the British Isles [13]. Blue whiting spawns at 300–600 m depth [84] and the eggs rise initially in the water column before they assumedly sink during mid stage development due to changes in osmoregulation [27]. Blue whiting eggs hatch at 200–600 m depth, and larvae rise towards the surface because of their lower specific gravity due to the loss of the heavy chorion. The changes in egg specific gravity through development as described for eggs of Cape hake [5], walleye pollock [28], sardine and anchovy [84], anchovy [29], and for Atlantic cod [27], and explained as changes in the osmoregulation [27,85], have the potential to keep eggs retained at mesopelagic depths also in a situation with decreasing salinity with depth when the egg buoyancy, *Δρ*, is very small. Under such a situation the eggs could rise during the initial stage, thereafter sink during the mid stages when they become heavier due to loss of water, and rise again during the final stages when the eggs become lighter due to restoration of the water balance. This could be an important mechanism for retaining eggs at mesopelagic depths in boreal and Arctic regions where the duration of the egg stage is very long. For example, Greenland halibut (*Reinhardtius hippoglossoides*) that spawns at 1000–1500 m depth in the Labrador Sea have mesopelagic eggs that reach the surface layers only after hatching [86,87]) even though the incubation time is more than one month depending on temperature [88].

## Discussion

The osmoregulation in teleost eggs, that keeps the internal salinity approximately constant independent of ambient salinity [7,17–20], gives the eggs some particular physical-biological attributes different from the major part of marine organisms, e.g. invertebrate plankton, where osmotic shrinkage from the high-saline environment is prevented by organic osmolytes [89]. These special attributes of teleost eggs imply that their *in-situ* buoyancy, *Δρ*, can be calculated at high precision based on laboratory measurements of the egg neutral buoyancy with reference to salinity alone [21] and on the hydrographic profile of the water column. This, in turn, gives the opportunity to model the vertical distribution of the eggs based on spawning depth, salinity profile and vertical mixing [11,12,30,31,57–61,68,71–73]. All of these citations relate to a water column of increasing salinity with depth which is the characteristic feature of coastal and shelf regions where most fish species spawn [32]. However, the major parts of world oceans, including coastal regions of the eastern boundary upwelling ecosystems, have a water column with decreasing salinity with depth. The theory of the present paper suggests that the fish species spawning mesopelagically under such conditions must have developed a special spawning strategy. Documentation of such a strategy is found for Cape hake in the Northern Benguela upwelling ecosystem [5], and for the blue whiting west of the British Isles [13]. Here we have analyzed the mechanisms linked to such kind of adaption, i.e. the physical structure of the water column and physico-chemical and physical-biological attributes of the eggs

The specific gravities, *ρ*, of the various basic egg components, the lipids [35–39] and the proteins [24,40,49] and the osmolarity of the water content of the [18–20] can be given at a relatively high precision. In addition, the volumes of basic components can be measured, such as the chorion volume, *V*_*ch*_, and volume of the perivitelline space, *V*_*periv*_, This enables us to analyze (by Eq 2) the relative contribution of each of the basic components to the overall egg specific gravity, *ρ*_*egg*_, and to analyze causes of change in *ρ*_*egg*_ throughout incubation [27]. It implies that the factors causing initial (i.e. at time of spawning/fertilization) variations in egg specific gravities within species, as well as between species, are the variations of volume fractions of the various components of the egg. For example, variation in the chorion volume fraction, (*V*_*ch*_/*V*_*egg*_)×100, ranging from 1.5 to 4.4%, is the most important factor for variation in egg specific gravity in Atlantic cod [27]. Moreover, the volume fraction of the perivitelline space, *V*_*periv*_/*V*_*egg*_, vary substantially between species. While (*V*_*periv*_/*V*_*egg*_)×100 in Atlantic cod varies between 9 and 18% [27], it may reach 90% in the sardine egg [22,30]. Eq 2 is, therefore, a useful tool to analyze causes of changes in *ρ*_*egg*_. However, when it comes to absolute accuracy, only the density gradient column [21] meets the demands for calculating the precise egg buoyancy in the ocean needed to model the vertical distribution.

The estimates of egg volumetric thermal expansion, *α*_*v egg*_, by Eq 3 and presented in Table 2 confirm the earlier assumption [7] and observations [22] that for most practical purposes buoyancy in fish eggs is related to salinity alone and nearly insensitive to temperature variations. This is caused by the combination 3.5 times *α*_*v*_ of lipids and proteins and 0.7 times *α*_*v*_ of the water content compared to the *α*_*v*_ of ambient seawater. The calculations across the four different species under a variety of ambient hydrographic conditions demonstrate the robustness of this conclusion. Finally, it should be emphasized that the literature cited on thermal expansion coefficients on proteins are not specifically from proteins in fish egg. We, therefore, encourage investigations on fish egg proteins, particularly the differences between the hardened proteins of the chorion and the softer proteins of the yolk and embryo. Also, data on the fractional contribution of the lipids and proteins in the yolk and embryo [18] show that the lipid/protein ratio varies considerable among species, and additional data on individual species is needed in order to fully understand the mechanisms of variations in egg specific gravity.

The confirmation above that salinity is the only significant determinant for *in-situ* buoyancy, *Δρ*, in teleost eggs, even over a large range of vertical temperature variations (~10°C), leads to the present development of vertical distribution of mesopelagic eggs as illustrated in Figs 4 and 5. This states that steady-state vertical egg distributions are not a possible solution. It further implies that field sampling of the vertical distributions of such eggs gives little meaning unless they are also stage-determined as a function of depth, such that the distributions can be interpreted the correct dynamical framework. The concept of non-stationary vertical distribution of mesopelagic eggs under decreasing vertical salinity with depth also leads to define the *critical spawning depth* for an individual egg. That is equal to the depth level where *in-situ* ambient salinity equals the neutral buoyancy salinity of the individual egg. Spawning below this critical depth will cause the egg to sink out of the water column and become lost for recruitment. Since a spawning population produce eggs over a certain range of buoyancies (see Figs 2 and 4) a *critical spawning depth layer* must be defined on the population level. For the actual example given in the present paper for Cape hake eggs in the Northern Benguela upwelling ecosystem [5] the critical spawning depth layer will be around 50 m thick extending from about 400 to 450 m depth. However, taking into account the existence of internal breaking waves that might throw an egg spawned above the critical depth to larger depths would indicate an extra “security zone” above for sustainable spawning. Such internal breaking waves have been observed in the Northern Benguela and near the critical spawning depth layer for Cape hake. Strong temperature fluctuations at 450 m depth was observed by Monteiro *et al*. (2005) [90] and associated with internal tidal oscillations. Similarly, Mohrholz *et al*. (2014) [91] observed high level of turbulent kinetic energy (TKE) and low static stability (low Brunt-Väisälä frequency) above the bottom at depth between 350 and 400 m depth, also an indication of internal breaking waves. It is remarkable to note that the Cape hake spawn at depth between 200 and 350 m, hence, above the here defined critical spawning layer and probably also out of reach for the major region of internal breaking waves.

It can be questioned whether the depth selected by spawning Cape hake in the Northern Benguela is an adaptation caused by pure selection, i.e. those eggs that are spawned below the critical depth layer will not recruit to the population, or whether the spawning fish have some kind of sensing system that can detect the ambient salinity to an extremely high accuracy. If so, it has to be equal to the accuracy level of a high-precision CTD. If the adaptation is by selection it could be possible that the occurrence of anomalous salinity structures will contribute to recruitment variability. However, the salinity structure in the Benguela upwelling ecosystem is very stable between years. If the spawning fish has some kind of precise salinity sensory system, the fish has a robust system to counteract potential recruitment variability caused by egg sink out and larval loss to the hostile conditions at deeper depths.

Impacts of climate change on marine ecosystem comprise the influence of changes in temperature, wind-induced mixing, stratification, current pattern, light conditions, oxygen and acidification [33]. Particularly, the phytoplankton and zooplankton are influenced by a diverse set of climate variables. However, buoyancy in marine fish eggs will additionally be influenced by the change in salinity of the world oceans. In the subtropical Atlantic, including the Canary and the Benguela EBUEs, the sea surface salinity (SSS) has increased by 0.2–0.4 over the recent 60 years due to increased evaporation over precipitation, while in most over the of the western subtropical Pacific SSS has decreased by about 0.4 over the same period [92]. The surface effect extends down to about 500 m depth. Such salinity changes have the potential to change egg buoyancy and rising speed of the eggs non-stationary mesopelagic eggs, as well as change the critical spawning depth. For the case of Cape hake eggs in the Northern Benguela the increased salinity would have increased the rising speed of the eggs and lowered the critical spawning depth over the recent 60 years. For such eggs in the western tropical Pacific the opposite would have occurred: decreased rising speed of the eggs and made critical spawning depth shallower.

The vertical distributions of fish eggs across the world oceans display a wide variety of distributions and adaptations by selection of spawning sites and evolvement of egg buoyancies. *Spawning depth*, *hydrographic profile*, *egg specific gravity*, and *egg stage* are key information when investigating vertical egg distributions in the field. For most of the reported observations on vertical distribution of fish eggs additional information about egg specific gravity does not exist. For many reported egg surveys hydrographic profiles, including salinity, are also missing. In addition, information about stage-dependent vertical egg distributions and information about spawning depths are often missing. Such additional information is necessary in order to understand and predict vertical distribution and movement of the eggs and to understand how spawning behavior and vertical distributions develop under the future changing climate.

## Concluding Remarks

The mechanistic understanding of egg specific gravity and the *in-situ* buoyancy linked to the vertical structure of salinity in the ocean are key factors to understand and predict vertical movement and vertical distribution of fish eggs in the water column. This, in turn, constrains the horizontal transport and dispersion of the offspring. The regions and vertical strata occupied by fish eggs and larvae determine their exposure to predators and the opportunities for the larvae to feed. We have in this paper shown that the conditions for vertical distribution of eggs and their vertical transport are basically different for the regions of the world oceans were salinity increases with depth, largely the coastal regions, and the major areas of the oceans where salinity decreases with depth. Understanding the great variety of spawning adaptations and the resulting vertical distributions of the eggs across species and ecosystems are the initial needed approach to model the pelagic offspring transport from the time of spawning until it recruits to the fish stock, and to understand how climate change will impacts early life history of fish.
